# Medium-term retention and household diffusion of basic life support skills after a school-wide educational intervention: PLANIFICARCP PROJECT

**DOI:** 10.1016/j.resplu.2026.101279

**Published:** 2026-02-19

**Authors:** Alejandro Romero-Linares, Francisco M. Parrilla-Ruiz, Gerardo Gómez-Moreno, Ana Carrasco-Cáliz, Antonio Cárdenas-Cruz

**Affiliations:** a Department of Medicine, University of Granada, Internal Medicine Service, Section of Pneumology, Hospital Santa Ana de Motril, Granada, Spain; b Doctoral Candidate Doctoral Programme in Clinical Medicine and Public Health, University of Granada, CriticalLab CTS 609 Research Group, Spain; c Department of Medicine, University of Granada, Emergency Department, University Hospital Clínico San Cecilio, Granada, Spain; d IBS TEC 23 Research Group, IS Carlos III, CriticalLab CTS 609 Research Group, Spain; e Department of Stomatology, University of Granada, School of Dentistry, CTS 654 Research Group, Granada, Spain; f Department of Intensive Care Medicine, University Hospital Virgen de las Nieves, Granada, Spain; g Department of Medicine, University of Granada, Department of Intensive Care Medicine, University Hospital Virgen de las Nieves, Granada, Spain; h IP Research Group TEC 23, IBS Granada, IS Carlos III, Director of the CriticalLab CTS 609 Research Group, Spain

**Keywords:** Basic life support, Cardiopulmonary resuscitation education, School-based training, Skill retention, Community preparedness

## Abstract

**Background and aim:**

Early bystander intervention is a key determinant of survival after out-of-hospital cardiac arrest, and school-based cardiopulmonary resuscitation (CPR) training is widely recommended to strengthen community response. However, evidence on medium-term retention of procedural skills and on the diffusion of basic life support (BLS) knowledge from trained students to their household environment remains limited. The aim of this study was to assess medium-term retention of procedural BLS competencies in schoolchildren following a structured educational intervention, and to evaluate the diffusion of BLS knowledge and perceived capacity to act to family members.

**Methods:**

This study evaluated students from primary, secondary, and high school and a voluntary subsample of family members in a school in Granada (Spain). Sociodemographic characteristics and cognitive and attitudinal variables were collected using an anonymized online questionnaire. Procedural basic life support (BLS) competencies were assessed approximately four months after the educational intervention through face-to-face simulation using a structured rubric applied by external evaluators trained in BLS. Household diffusion was evaluated through family-reported outcomes, including discussion of the training experience at home and perceived capacity to act in an emergency.

**Results:**

The intervention included 683 students and 196 family members. At medium-term follow-up, students showed high procedural performance in key BLS actions, including high rates of adequate chest compression quality (89.3%), correct AED pad placement (81.8%), and safe shock delivery (91.9%). Household diffusion was substantial, with most relatives reporting discussion of the training experience at home and approximately half reporting active teaching attempts by the student. Relatives’ perceived capacity to act increased markedly.

**Conclusion:**

A structured, school-wide BLS intervention delivered in the school setting supports sustained procedural competence in schoolchildren and facilitates meaningful diffusion of resuscitation knowledge and confidence to the household environment. These findings reinforce the role of schools as strategic platforms for scalable interventions aimed at strengthening community preparedness for out-of-hospital cardiac arrest.

## Introduction

Out-of-hospital cardiac arrest (OHCA) remains a leading cause of mortality worldwide, with survival critically dependent on early recognition, prompt activation of emergency medical services, and immediate initiation of basic life support (BLS) by bystanders. A substantial proportion of OHCAs occur in the home environment, where the presence of trained laypersons is often the only determinant of early life-saving actions.^1^, ^2^ In response to this public health challenge, international organisations and resuscitation councils strongly recommend widespread population training in cardiopulmonary resuscitation (CPR), with particular emphasis on integrating BLS education into compulsory school curricula. School-based CPR training has been identified as a cost-effective and sustainable strategy to increase the proportion of trained citizens and to normalise resuscitation skills at a societal level.^1^, ^3^ International implementation efforts strongly support school-based resuscitation education. In particular, the ‘KIDS SAVE LIVES’ initiative, supported by the International Liaison Committee on Resuscitation (ILCOR) and endorsed by the World Health Organisation, promotes the systematic inclusion of cardiopulmonary resuscitation training in school curricula to increase bystander CPR rates and strengthen community preparedness for out-of-hospital cardiac arrest.^4^, ^5^

In Spain, as in many other countries, legislative and curricular frameworks include references to BLS and automated external defibrillator (AED) training within primary and secondary education. However, a persistent gap exists between formal curricular inclusion and real-world implementation. This gap is characterised by heterogeneity in teaching methodologies, limited availability of trained instructors, variability in practical exposure, and a lack of standardised assessment of acquired competencias.^6^, ^7^ Beyond immediate skill acquisition, two critical but less frequently evaluated dimensions of school-based CPR training are medium-term skill retention and knowledge diffusion beyond the classroom**.** Retention of psychomotor CPR skills tends to decay over time, and most studies focus on short-term post-training assessments. Even fewer investigations explore whether trained students act as vectors of knowledge transmission to their families, despite the fact that households represent the most frequent setting for OHCA events.^3^, ^8^, ^9^ The present study addresses these gaps by evaluating a structured school-wide BLS intervention conducted in a Spanish educational community. Specifically, it examines (i) the medium-term retention of procedural BLS competencies assessed approximately four months after training using standardised simulation-based rubrics, and (ii) the extent to which training effects diffuse to the household, as reported by family members, including changes in perceived capacity to act in an emergency. The aim was to evaluate medium-term retention of BLS procedural competencies in schoolchildren following a structured educational intervention, and to quantify the real diffusion of BLS knowledge and confidence from students to their household environment.

## Methods

### Study design and setting

We conducted a school-based educational intervention in Granada (Spain) delivered within the facilities of a single educational centre. All study activities (training and evaluation phases) were carried out in spaces provided by the school. The study received a favourable opinion from the Research Ethics Committee of the University of Granada (Spain) (registry 3874/CEIH/2023). Authorizations for study development and results dissemination were obtained from the school management team, along with individual authorizations by legal representation for each student. The study followed the principles of the Declaration of Helsinki and used anonymized coding to safeguard participant confidentiality and data protection compliance.

### Participants

**Fig. 1:**
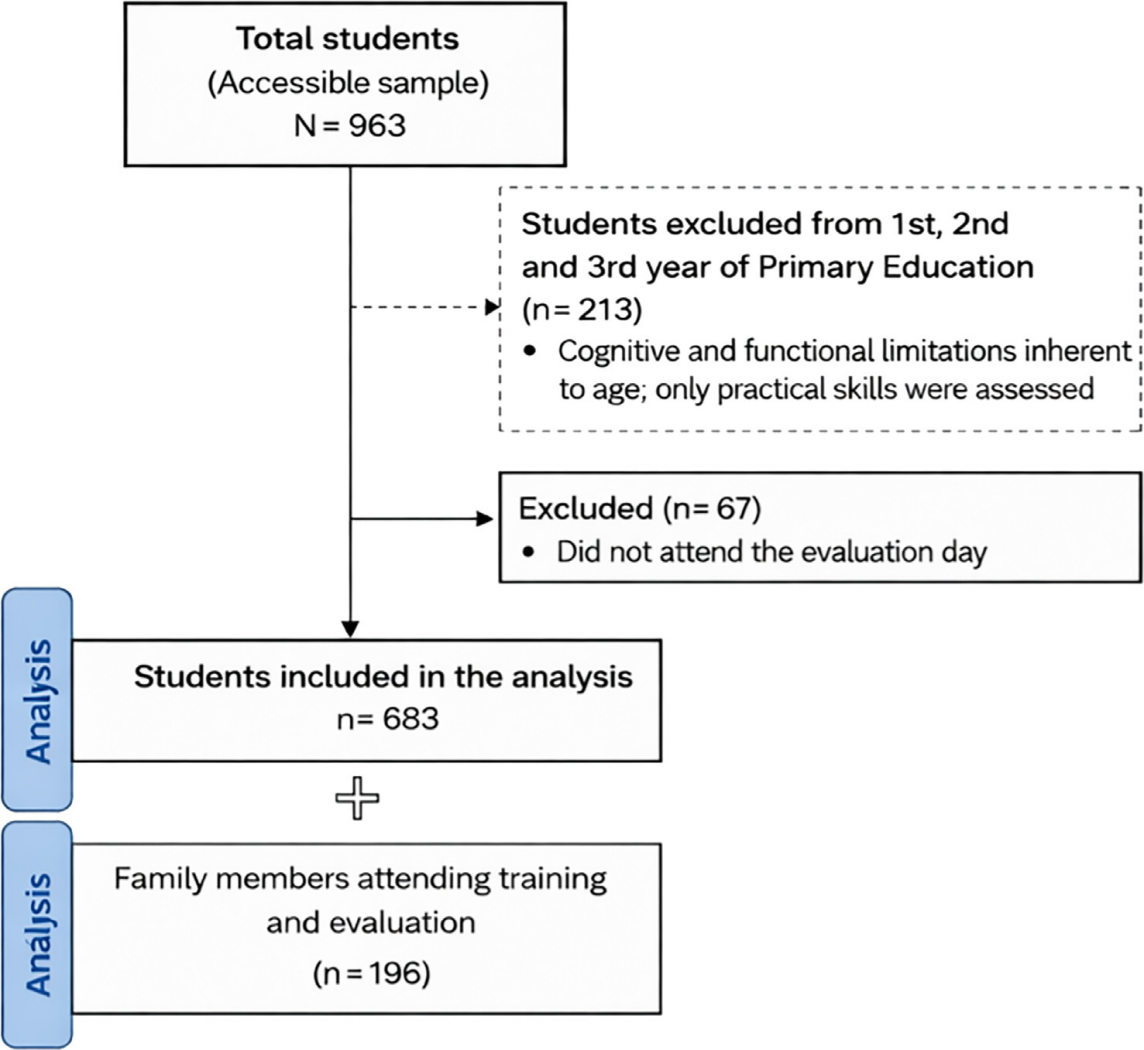
**Study flowchart**. Flow diagram showing participant inclusion, exclusion, and follow-up throughout the three phases of the study (pre-intervention, immediate post-intervention, and delayed evaluation).

The study population comprised the educational community of La Inmaculada HH Maristas (Granada, Spain), including students registered during the 2023/2024 academic year, spanning ages 5–19 years (from 1st year of primary education to 2nd year of high school). The school included 36 classes (three classes per grade), with a total enrolment of 998 students. After applying exclusion criteria across the study phases, the total number of students ultimately evaluated was 683 (Fig. 1). Participants included 683 students aged 8–19 years. The distribution by academic grade was: Primary Education (*n* = 220): 4th grade (*n* = 74), 5th grade (*n* = 74), and 6th grade (*n* = 72); Secondary Education (*n* = 336): 1st year (*n* = 94), 2nd year (*n* = 85), 3rd year (*n* = 74), and 4th year (*n* = 83); High School/Bachillerato (*n* = 127): 1st year (*n* = 66) and 2nd year (*n* = 61). A voluntary household component was also included: family members/cohabitants of participating students could participate in the family training/evaluation and complete the family questionnaire. In this family component, ‘direct observation of practice’ referred to the structured, in-person assessment of practical skills during voluntary school-based sessions. Family participants performed BLS/AED actions on manikins and AED trainers in a simulated setting, and their performance was directly observed and scored in real time by trained evaluators using a standardised rubric. This term does not refer to home-based observation or remote monitoring. Prior to enrolment, no formal or structured BLS training had been provided to participants; consequently, this intervention constituted their first exposure to structured BLS education. Students were eligible if they: (i) were enrolled in primary, secondary, or high school at the participating centre during the academic year of the intervention; (ii) participated in the school-based BLS training programme; and (iii) were available for the medium-term practical skills assessment (∼4 months). For the household analysis, eligible participants were family members/cohabitants of participating students who voluntarily completed the family questionnaire. Students were excluded if they were absent during evaluation sessions or could not complete the theoretical assessment due to technical issues. Additionally, incomplete or invalid questionnaire data preventing linkage across phases led to exclusion from analyses requiring longitudinal linkage. Family members were excluded from household diffusion analyses if they did not complete the household diffusion questionnaire.

### Educational intervention and evaluation framework

The educational programme combined adaptive teaching approaches, clinical simulation and playful learning strategies, aiming to promote active engagement across age groups.

The programme was implemented as a whole-school intervention with age-adapted pedagogical strategies. Primary school students received a playful, experiential approach focused on engagement and basic sequence recognition; secondary students followed a mixed model combining structured explanation and deliberate practice of the full BLS/AED algorithm; and high-school students participated in more simulation-oriented training with higher technical performance requirements. Across all age groups, training was delivered by certified instructors and procedural skills were assessed using a standardised rubric by external evaluators The evaluation strategy was designed to capture three competency domains (cognitive, attitudinal, and procedural) aligned with the staged structure of the project. Training delivery relied on University of Granada (Spain) medical students accredited in BLS and defibrillation, while procedural skills assessment was performed by external evaluators not involved in the teaching phase and trained in rubric-based assessment of procedural competencies.

### Data collection

Data collection was conducted sequentially, aligned with the study phases. Sociodemographics and cognitive/attitudinal competencies were collected during the first weeks of March 2024 using an anonymized online questionnaire administered in the classroom under researcher supervision. Microsoft Forms® was used, linked to an-Excel file that automatically recorded responses for subsequent analysis. To ensure confidentiality and enable linkage across phases, each student was assigned an anonymous identification code, allowing phase-to-phase matching without storing personal identifiers. Procedural competencies were assessed face-to-face approximately four months after the training intervention (late April 2024). External evaluators accredited in BLS observed each student individually in a simulated scenario and scored performance using a structured rubric with item-specific steps corresponding to the essential BLS algorithm, including AED-related actions. Household diffusion was assessed through family-reported outcomes addressing whether the student discussed the training experience at home and whether the student attempted to explain/teach the manoeuvres. Household diffusion was not mandated as a homework task; it was evaluated as a real-world post-training behaviour, and the household component was voluntary. Perceived capacity to act (family members) was evaluated pre/post using ordinal response categories ranging from “incapable” to “very capable”. In the family component, evaluation combined online testing and direct observation of practice. Simulation material included multiple manikin models and AED trainers: Multi Man® (Ambu A/S, Ballerup, Denmark), RCP Brayden IM16-R adulto PRO® (Innosonian Inc., Seoul, South Korea), Heartstart FR2® (Royal Philips, Amsterdam, The Netherlands), and XFT-120C+Practice-Trainer® (Shenzhen XFT Medical Limited, Shenzhen, China).

### Outcomes

Primary outcomes were: (1) student procedural performance at ∼4 months in simulation, based on rubric-defined success in key actions (e.g., chest compressions, AED pad placement, safe shock delivery); and (2) real household diffusion (discussion at home; active teaching attempt). Secondary outcomes included changes in family members’ perceived capacity to act.

### Statistical analysis

Paired comparisons were assessed using Wilcoxon signed-rank tests, reporting Hodges–Lehmann estimators for median differences and Wilcoxon effect size r. Associations between categorical variables were evaluated using Chi-square or Fisher’s exact tests, with Cramér’s *V* as an association measure; adjusted standardised residuals were used to identify contributing categories. Sex comparisons were expressed using odds ratios (OR) with 95% confidence intervals. Statistical significance was set at *p* < 0.05. Analyses were performed using R (Mac).

## Results

The intervention reached a large school-based cohort including 683 students from primary, secondary, and high school levels. In addition, a voluntary subsample of 196 family members participated in the household component of the study. At approximately four months after the educational intervention, students showed high retained procedural performance in simulated BLS scenarios. Adequate overall technical quality of chest compressions was achieved by 89.3% of students (*n* = 610)**,** indicating preservation of the core psychomotor component of cardiopulmonary resuscitation. Correct AED pad placement was observed in 81.8% (*n* = 559)**,** and safe shock delivery was successfully performed by 91.9% (*n* = 628) (Table 1). Household diffusion of learning was substantial. Among participating relatives, 80.6% (*n* = 158/196) reported that the student discussed the training experience at home, and 51.0% (*n* = 100/196) reported that the student actively attempted to explain or teach BLS maneuvers. Patterns of diffusion differed by educational level. The likelihood of telling the experience at home varied significantly across school grades (*p* < 0.001; Cramér’s *V* = 0.20). Analysis of adjusted standardised residuals revealed a clear age-related trend: reporting the experience at home was significantly higher than expected in the final years of primary education (5th Grade residual = 2.88; 6th Grade residual = 1.98). Conversely, students in lower secondary education showed lower-than-expected rates of household diffusion, particularly in the 3rd year (residual = −3.12) and 4th year (residual = −2.23). The likelihood of telling the experience at home varied significantly across school grades (*p* < 0.001; Cramér’s *V* = 0.20**),** with higher-than-expected reporting in the final years of primary education. In contrast, teaching the maneuvers at home did not show statistically significant differences across grades (*p* = 0.07). Family attendance at the course also varied significantly by educational level (*p* < 0.001; Cramér’s *V* = 0.20**)** (Table 2). Sex-based comparisons showed that female students were more likely to report telling the experience at home (*p* = 0.03), with an odds ratio of 1.87 (95% CI 1.07–3.28) compared with male students, whereas differences in teaching at home by sex were not statistically significant (*p* = 0.178) (Table 2).

**Table 1:** Frequencies and percentages of performance level for each variable in the practical–procedural assessment (*n* = 683).

**Evaluated competency**	**No** **(*n*)**	**No** **(%)**	**Partial** **(*n*)**	**Partial** **(%)**	**Correct** **(*n*)**	**Correct** **(%)**
Checks responsiveness	91	13.3	95	13.9	497	72.8
Checks breathing	18	2.6	176	25.8	489	71.6
Calls for help and activates emergency number (112)	14	2	51	7.5	618	90.5
Requests an-AED	15	2.2	67	9.8	601	88
Performs chest compressions	2	0.3	71	10.4	610	89.3
Turns on and places AED pads	10	1.5	114	16.7	559	81.8
Delivers shock	6	0.9	49	7.2	628	91.9
Continues chest compressions	43	6.3	34	5	606	88.7
Correct algorithm sequence	66	9.7	–	–	617	90.3

**Table 2:** Association between school grade and information flow: *p*-value, Cramér’s *V*, and adjusted residuals for grades showing statistically significant associations, and adjusted residuals for grades showing statistically significant associations.

**Variable**	***p*-value**	**Cramér’s *V***	**Grades with significant residuals** **(*category: Yes*)**	**Residual values**
Attendance of a family member at the course	<0.001	0.20	2nd year of Lower Secondary Education	2.20
			3rd year of Lower Secondary Education	−4.42
			4th year of Primary Education *(trend)*	1.57

Telling the experience at home	<0.001	0.20	5th year of Primary Education	2.88
			6th year of Primary Education	1.98
			3rd year of Lower Secondary Education	−3.12
			4th year of Lower Secondary Education	−2.23

Relatives’ perceived capacity to respond to a cardiac arrest scenario improved markedly following the intervention. Before exposure, only 0.5% (*n* = 1) of relatives rated themselves as very capable, whereas this proportion increased to 65.3% (*n* = 128) after the intervention. Similarly, the proportion rating themselves as capable increased from 12.2% (*n* = 24) to 31.6% (*n* = 62)**,** and the lowest perceived capacity categories (incapable and poorly capable) were absent after the intervention. In addition, 95.4% of relatives reported that it was quite or very likely that they would further disseminate the acquired knowledge within their social environment (Table 3). Perceived capacity was strongly related to diffusion behaviours. Students’ self-perceived capacity was associated with both reporting the experience at home (*p* = 0.002; Cramér’s *V* = 0.146**)** and, more strongly, with teaching attempts at home (*p* < 0.001; Cramér’s *V* = 0.41) (Table 4). At the household level, relatives’ baseline self-perceived capacity was also associated with whether the student had previously taught them at home **(***p* = 0.043; Cramér’s *V* = 0.216). However, objective family performance did not differ according to prior teaching at home (cognitive score *p* = 0.871**;** practical score *p* = 0.708**)** (Table 5).

**Table 3:** Frequencies and percentages of perceived capacity before and after the intervention in the family subsample (*n* = 196).

**Perceived capacity level**	**Time point**	**Frequency (*n*)**	**Percentage (%)**
Incapable *(coded as 1)*	Before	71	36.2
	After	0	0

Poorly capable *(coded as 2)*	Before	38	19.4
	After	0	0

Somewhat capable *(coded as 3)*	Before	62	31.6
	After	6	3.1

Capable *(coded as 4)*	Before	24	12.2
	After	62	31.6

Very capable *(coded as 5)*	Before	1	0.5
	After	128	65.3

**Table 4:** Association between self-perceived capacity and willingness to report and teach at home: *p*-value, Cramér’s *V*, and adjusted residuals.

**Variable**	***p*-value**	**Cramér’s *V***	**Categories with significant residuals** ***(category 1 = Yes)***	**Residual values**
Willingness to report the experience at home	0.002	0.146	Very capable	0.09
			Capable	−1.22
			Poorly capable	0.55
			Incapable	−3.72

Willingness to teach at home	<0.001	0.41	Very capable	4.22
			Capable	1.67
			Poorly capable	−9.54
			Incapable	−4.09

**Table 5:** Association between relatives’ pre-training self-perceived capacity and prior teaching at home by the student: *p*-value, Cramér’s *V*, and adjusted residuals.

**Variable**	***p*-value**	**Cramér’s *V***	**Categories with significant residuals** ***(category: Not taught)***	**Residual values**
Relatives’ pre-training self-perceived capacity	0.043	0.216	Very capable	−0.98
			Capable	−2.51
			Somewhat capable	0.50
			Poorly capable	−0.58
			Incapable *(trend)*	1.85

## Discussion

This study shows that a structured, school-wide BLS programme can achieve high medium-term retention of key procedural competencies in schoolchildren and can extend its reach into households through measurable diffusion behaviours reported by family members. In the present intervention, more than four out of five students retained adequate chest compression quality and over 90% correctly performed AED-related actions at approximately four months post-training, supporting the concept of schools as high-yield settings to strengthen community readiness for out-of-hospital cardiac arrest (OHCA), particularly given that many OHCAs occur in the home environment and depend on immediate bystander response.^1^, ^10^, ^11^, ^12^

At approximately four months post-training, students maintained high performance in essential actions, including chest compression quality (89.3%) and AED-related steps such as pad placement (81.8%) and safe shock delivery (91.9%). This medium-term snapshot is especially relevant because psychomotor CPR skills are known to decay over time in the absence of refresher training, particularly among younger learners and when exposure is limited.^3^, ^8^, ^13^, ^14^ The sustained performance observed in this cohort may be related to several design features of the programme, including structured sessions adapted to educational level, a strong emphasis on hands-on practice through stations and simulations, and objective assessment using a standardised rubric applied by external evaluators trained in BLS. From an implementation perspective, these components are reproducible and align with current recommendations that advocate for consistent delivery and objective evaluation standards in school-based CPR education.^1^

A distinctive contribution of this work is the systematic evaluation of household diffusion from the relatives’ perspective. While previous initiatives frequently assume a “multiplier effect” whereby trained children influence family members, empirical confirmation using family-reported outcomes remains limited.^2^, ^9^ In the present study, 80.6% of relatives reported that the student discussed the BLS training experience at home, and 51.0% reported active teaching attempts by the student. These results indicate that diffusion is not merely hypothetical but occurs in real-life household settings. This observation is particularly relevant for OHCA preparedness, as the home is a common location for cardiac arrest events and family members are often the only immediate responders before emergency medical services arrive.^1^, ^15^, ^16^, ^17^ Our findings are consistent with the rationale of the ‘KIDS SAVE LIVES’ initiative, which frames children not only as future potential rescuers but also as multipliers who can disseminate resuscitation knowledge within their families and communities. The substantial household diffusion observed (discussion at home and teach-back attempts reported by relatives) provides empirical support for this multiplier pathway and reinforces schools as scalable platforms for public health strategies aimed at improving bystander response and early defibrillation readiness.

Recent qualitative research has provided valuable insights into how basic life support training can be optimised from the learners’ and teachers’ perspectives.^18^ This study explored how children and teachers would modify a basic life support (BLS) course designed by specialists. This qualitative study used video-stimulated recall interviews following a standard 90-minute training session in adolescents aged 12–16 years. The authors provided very specific recommendations, including adapting language and audiovisual materials to the learners’ reality, increasing emotional engagement and action, limiting group size in the final scenario, and improving curricular integration.^18^ These findings contribute to optimising pedagogical design from both the end-user perspective (students) and the educational agent (teachers). PLANIFICARCP, in contrast, provides quantitative evidence on skill retention and knowledge diffusion, together with a design that already incorporates adaptation across educational levels. The complementarity between both approaches is clear, as the qualitative study informs how the intervention can be improved, whereas our study allows assessment of whether learning is maintained over time and transferred to the home environment.

Relatives’ perceived capacity to act improved markedly following the intervention, with the proportion rating themselves as “very capable” increasing from 0.5% before exposure to 65.3% afterwards, and the complete disappearance of low perceived capacity categories. Confidence and willingness to intervene are recognised determinants of bystander CPR and may influence whether individuals translate knowledge into action during emergencies.^2^, ^19^, ^20^ In parallel, the observed associations between perceived capacity and diffusion behaviours, particularly the strong relationship between students’ self-perceived capacity and active teaching at home (Cramér’s *V* = 0.41; *p* < 0.001), support the notion that self-efficacy may facilitate communication and informal teaching within households, reinforcing the social transmission pathway of resuscitation knowledge.

From a public health perspective, these findings support educational strategies that view children not only as future potential rescuers but also as active agents capable of catalysing household-level engagement. Embedding BLS education within schools may therefore contribute to a cumulative effect on community preparedness, complementing broader public-access defibrillation and lay responder strategies promoted by international councils. A practical implication of this work is that effective programmes should combine age-adapted pedagogy, structured practical training, and objective assessment, while also considering simple mechanisms, such as encouraging discussion or teach-back at home, that may facilitate secondary diffusion beyond the classroom.^1^, ^2^

One study evaluated a blended learning programme for schoolchildren combining brief theoretical sessions, manikin-based practice, and a virtual reality component.^21^ Using a before–after design, it assessed immediate changes in attitudes and knowledge, as well as acceptability of the technological component. In a sample mainly composed of upper secondary school students, willingness to intervene in cardiac arrest increased markedly after the course, with particularly high perceived realism and engagement among younger participants.^21^ Compared with PLANIFICARCP, both approaches aim to improve community preparedness; however, while this study provides evidence of immediate change (knowledge, attitudes, and technology acceptability),^21^ PLANIFICARCP demonstrates medium-term retention of procedural performance and transfer to the home environment. Together, these findings are complementary: immediate acceptability and motivation facilitate implementation, whereas retention and diffusion determine population-level impact.

Strengths of this study include the large school-based cohort, the structured programme delivered across multiple educational levels, and the objective assessment of procedural skills using trained external evaluators and a standardised rubric. Several limitations must also be acknowledged. The household component relied on a voluntary subsample of family members, which may introduce self-selection bias, as participating families could be more health-motivated or engaged with school activities. Consequently, household findings should be interpreted as pragmatic implementation outcomes within the participating subsample rather than as population-level estimates. Although the associations are unlikely due to chance, their strength is modest and should be interpreted considering the multifactorial nature of the phenomenon studied. Procedural performance was assessed using manikin-based simulation, which cannot fully replicate the stress, environmental constraints, and emotional context of real-life OHCA situations. The absence of a non-trained control group limits causal inference, although ethical considerations often preclude withholding potentially life-saving education in school settings. Follow-up was limited to a medium-term period of approximately four months; longer-term durability of skills and diffusion effects remains to be established, and future research should evaluate whether periodic refresher training optimises sustained performance and household-level impact over time.^3^, ^8^ Procedural performance was assessed using a structured rubric aligned with the BLS/AED algorithm and applied by trained external evaluators. While this tool has strong content validity, it was not designed as a psychometrically validated scale; future studies should incorporate formal inter-rater reliability analyses (e.g., kappa or intraclass correlation coefficients).

## Conclusion

This school-wide educational intervention demonstrates that structured basic life support (BLS) training delivered in the school setting can achieve meaningful medium-term retention of key procedural competencies in schoolchildren and promote substantial diffusion of resuscitation knowledge to the household environment. Beyond skill retention, the findings highlight the potential of students to act as conduits for increasing confidence and readiness to respond among family members. These results support the role of schools as strategic platforms for scalable BLS education with relevance for community preparedness for out-of-hospital cardiac arrest.

## Sources of funding

This research did not receive any specific grant from funding agencies in the public, commercial, or not-for-profit sectors.

## Disclosures

The authors declare that they have no relevant financial or non-financial relationships to disclose.

## CRediT authorship contribution statement

**Alejandro Romero-Linares:** Writing – review & editing, Visualization, Validation, Supervision, Resources, Methodology, Investigation, Formal analysis. **Francisco M. Parrilla-Ruiz:** Writing – review & editing, Writing – original draft, Visualization, Validation, Supervision, Software, Resources, Methodology, Investigation, Formal analysis, Data curation, Conceptualization. **Gerardo Gómez-Moreno:** Writing – review & editing, Writing – original draft, Visualization, Validation, Supervision, Software, Resources, Methodology, Investigation, Formal analysis, Data curation, Conceptualization. **Ana Carrasco-Cáliz:** Investigation, Conceptualization. **Antonio Cárdenas-Cruz:** Writing – review & editing, Writing – original draft, Visualization, Validation, Supervision, Software, Resources, Methodology, Investigation, Formal analysis, Conceptualization.

## Declaration of competing interest

The authors declare that they have no known competing financial interests or personal relationships that could have appeared to influence the work reported in this paper.

## Data Availability

The datasets generated and analysed during the current study are not publicly available due to ethical and privacy considerations related to the participation of minors. Data may be made available from the corresponding author upon reasonable request and subject to approval by the relevant institutional ethics committee.
